# Supplementary material for: Natural Language Processing for Information Extraction of Gastric Diseases and Its Application in Large-Scale Clinical Research
Source: J Clin Med. 2022 May 24;11(11):2967. doi: 10.3390/jcm11112967 (PMC9181010; doi:10.3390/jcm11112967)
Supplement: Supplementary file 1 [file jcm-11-02967-s001.zip › updated Supplementary_Materials.pdf]

Supplementary Table S1. Summary of dictionary for gastric disease.

Variables	Impression	Findings
Location		
Antrum	-	antrum, angle, pylorus, pyloric orifice, pyloric sphincter
Body	-	body, high body, mid body, low body
Fundus	-	fundus, cardia, gastroesophageal junction
Chronic gastric		
Atrophic Gastritis	atrophic	thin, atrophy, vascularity, hyperemia, nodular, grey, pale, granular, vessel
Intestinal Metaplasia	metaplasia	nodular, granular grey, white, hyperemia, velvety
Superficial Gastritis	superficial	hyperemia, edema, hemorrhage, erythematous, congestion, punctate
Erosive Gastritis	erosive, verrucous	erosion, verruca, elevation, depression, hyperemia
Follicular Gastritis	follicular	follicular, granular, nodular, chicken, goose, protrusion
Other gastric diseases		
Ulcer	ulcer, benign gastric ulcer	ulcer, depression, yellowish, converge
Polyp	polyp	polyp, elevation, raised
SMT	submucosal tumor, subepithelial tumor	protrude, nodule, submucosal tumor, elevation, subepithelial tumor,
Dysplasia *	-	tubular adenoma

Cancer †	-	neuroendocrine tumor, adenocarcinoma, carcinoma, lymphoma with malt, maltoma

* Dysplasia includes tubular adenoma with low grade dysplasia. † Cancer includes carcinoma, neuroendocrine tumor, maltoma, and lymphoma with malt. SMT: submucosal tumor.
